# Split-gated point-contact for electrostatic confinement of transport in MoS_2_/h-BN hybrid structures

**DOI:** 10.1038/s41598-017-00857-7

**Published:** 2017-04-07

**Authors:** Chithra H. Sharma, Madhu Thalakulam

**Affiliations:** School of Physics, Indian Institute of Science Education and Research Thiruvananthapuram, 695016 Kerala, India

## Abstract

Electrostatically defined nanoscale devices on two-dimensional semiconductor heterostructures are the building blocks of various quantum electrical circuits. Owing to its atomically flat interfaces and the inherent two-dimensional nature, van der Waals heterostructures hold the advantage of large-scale uniformity, flexibility and portability over the conventional bulk semiconductor heterostructures. In this letter we show the operation of a split-gate defined point contact device on a MoS_2_/h-BN heterostructure, the first step towards realizing electrostatically gated quantum circuits on van der Waals semiconductors. By controlling the voltage on the split-gate we are able to control and confine the electron flow in the device leading to the formation of the point contact. The formation of the point contact in our device is elucidated by the three characteristic regimes observed in the pinch-off curve; transport similar to the conventional FET, electrostatically confined transport and the tunneling dominated transport. We explore the role of the carrier concentration and the drain-source voltages on the pinch-off characteristics. We are able to tune the pinch-off characteristics by varying the back-gate voltage at temperatures ranging from 4 K to 300 K.

## Introduction

Electrostatic gating is a versatile technique to engineer the electron flow in 2D systems. Gate defined quantum point contacts (QPC)^1, 2^ and quantum dots^3^ are the basic building blocks of potential devices for quantum information^4^, quantum metrology^5^ and charge sensing applications^6–8^. Spatial variation in the dopant density, interface roughness and the well width can greatly modify the operating conditions of devices realized on bulk semiconductor heterostructures. As a result, large-scale uniformity in the operating conditions and device performance could be technically challenging. Heterostructures combining van der Waals (vW) semiconductors and insulators^9–13^, with its atomically flat interfaces and the two-dimensional nature across the entire device area, could provide the required large-scale uniformity. In addition, the substrate independence of vW systems could pave way for flexible and transferable quantum circuits.

Electrostatically defined quantum dots on bilayer graphene-hexagonal boron nitride (h-BN) devices^14^ and split-gate defined QPCs on Graphene-Al_2_O_3_ structures have been demonstrated^15^. In contrast to graphene, molybdenum disulfide (MoS_2_)^16^ offers superior electrostatically tunable devices owing to the presence of a sizable band gap. Dielectric encapsulation have improved the electron mobility and the on-off ratio in MoS_2_ transistors^17–22^. Compared to conventional oxide interfaces MoS_2_/h-BN devices offer better electrical properties and stability owing to cleaner and flatter interfeces^22, 23^. Quantum transport phenomena such as quantum Hall effect and Shubnikov-de Haas oscillations are observed in MoS_2_/h-BN devices^24^. In addition, recently, single electron transport has been reported on MoS_2_^25^ making it a potential candidate for hosting future quantum circuits. Dual gating on h-BN/MoS_2_ has offered better and controllable devices^9, 22^. A natural continuation in this regard would be the electrostatic shaping and control of transport on MoS_2_ based vW heterostructure for nano-electronic applications.

In this report we demonstrate the electrostatic shaping and confinement of electron transport on a MoS_2_/h-BN heterostructure using a split-gate defined point-contact. The MoS_2_/h-BN heterostructure is formed by aligned transfer of layers of MoS_2_ and h-BN on a SiO_2_/Si wafer using a home-built micro-positioning system. Standard electron-beam lithography is used to define the drain and the source contacts and, the split-gate defining the point-contact. The electron flow in the device is controlled and confined by varying the voltage on the split-gate in a temperature regime between 4 K and 300 K. In contrast to the conventional top-gated FETs the conductance of our devices as a function of split-gate voltage exhibit transport regimes (i) that of a conventional FET, (ii) formation of the point contact and (iii) the tunneling dominated transport. By reducing the split-gate voltage one can sequentially access these regimes and eventually shutdown the transport by pinching-off the constriction. The heavily doped silicon back-gate is used for tuning the carrier concentration in the MoS_2_ layer. We also study the dependence of the point-contact formation on the drain-source voltage and back-gate voltage.

## Results and Discussions

MoS_2_ flakes, mechanically exfoliated from bulk crystals are transferred onto a clean 300 nm SiO_2_/Si substrate using PDMS dry transfer technique^26^. The drain and the source contacts are defined by electron-beam lithography followed by Cr/Au metallization. Thin flakes of h-BN were carefully placed on top of the MoS_2_ flake using the PDMS dry transfer technique using a micro-positioning setup^26^. The PDMS dry transfer technique is known to leave fewer residues compared to direct exfoliation using scotch tape or wet transfer techniques^27^. Wet transfer techniques may also cause wrinkles on the flakes affecting the flatness and device performances. The Raman spectra to inspect the structural quality of the samples was taken using Horiba Xplora Plus spectrometer with 532 nm excitation. The split-gate defining the point-contact is fabricated on top of the h-BN using electron-beam lithography and Cr/Au metallization. The heavily doped underlying Si substrate is used as the global back-gate for controlling the carrier concentration in the MoS_2_ layer. All electrical measurements are performed in a 4 K–300 K variable temperature cryostat in high vacuum (<10^−6 ^mbar) unlit environment.

**Figure 1:**
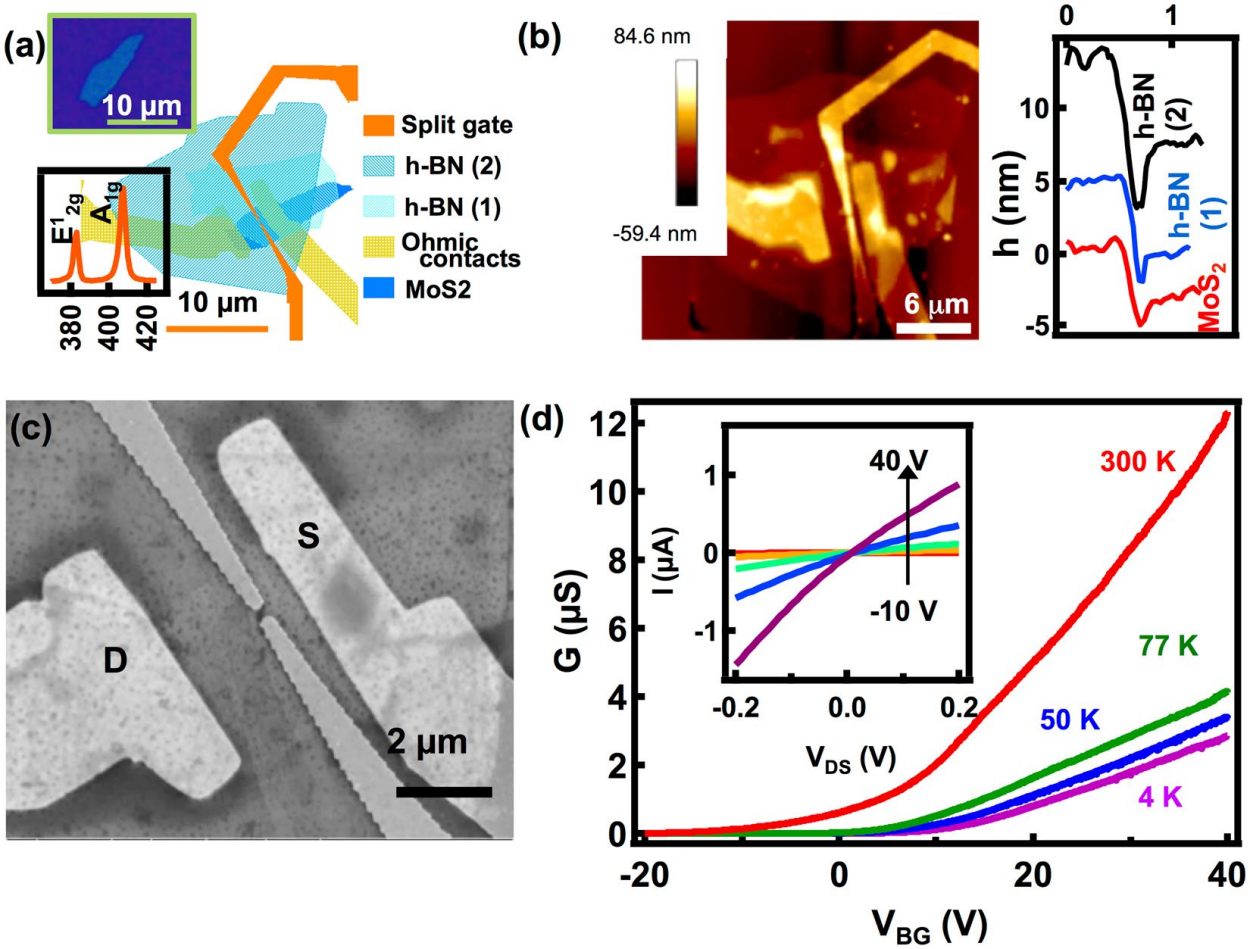
(**a**) A sketch of the device drawn to scale, with all the layers labelled. The top inset shows the optical image of the MoS_2_ flake and the bottom inset shows the Raman spectra of the MoS_2_ showing characteristic Raman peaks. (**b**) Left: AFM image of the device. Right: AFM height profiles of the MoS_2_, h-BN (1) and h-BN (2) flakes showing thickness of 3.5 nm, 5 nm and 6 nm respectively. (**c**) SEM image of the device showing the point-contact with a lithographic length of 280 nm and width 220 nm. The drain and source contacts are marked as D and S respectively. (**d**) Conductance versus back-gate voltage at four different temperatures. The inset shows I-V characteristics at 300 K, for various back gate voltages.

Figure 1(a) shows a sketch of the device drawn to scale. The top inset shows the optical image of the five-layer MoS_2_ flake (thickness 3.5 nm) on which the device is made. The optical and SEM image of the complete device and a diagram showing the stacking scheme of the heterostructure is given in Supplementary Information S1. From the optical and SEM images, we infer that sample is flat and free of any wrinkles. The Raman spectra shown in the bottom inset exhibit the characteristic E^1^_2g_ and A_1g_ peaks of MoS_2_ verifying the structural quality of the sample. Figure 1(b) – left panel shows the AFM image of the final device. We estimate the thickness of the MoS_2_ and the h-BN flakes from the AFM line profiles shown in the right panel. We have used two h-BN flakes with a total thickness of ~11 nm (5 nm + 6 nm) to ensure the complete coverage of the underlying MoS_2_ flake. The thickness of the MoS_2_ layer and the h-BN layers are extracted from the AFM height profiles shown in Supplementary Information S2. Figure 1(c) shows the scanning electron microscope (SEM) image of the device showing the drain and the source contacts and the split-gate defining the point-contact. The lithographic dimensions of the point-contact constriction are 280 nm in length and 220 nm in width.

Figure 1(d) shows the conductance of the MoS_2_ flake versus back-gate voltage (V_BG_) for various temperatures, from 300 K to 4 K with the split-gate grounded. The inset shows I-V characteristics of the device at 300 K for various V_BG_ values. The I-V characteristics and the conductance versus V_BG_ traces show that the sample is n-type. The threshold voltage estimated from the linear extrapolation of the conductance traces for all the temperatures are around V_BG_ ≈ 0 V and, we do not find any significant shift in the threshold voltage as the temperature is lowered, suggesting a lower trap density in our sample^19^. This also suggests that the dry transfer process employed here has maintained the sample and interface qualities and, the fabrication processes did not noticeably degrade the device performance.

**Figure 2:**
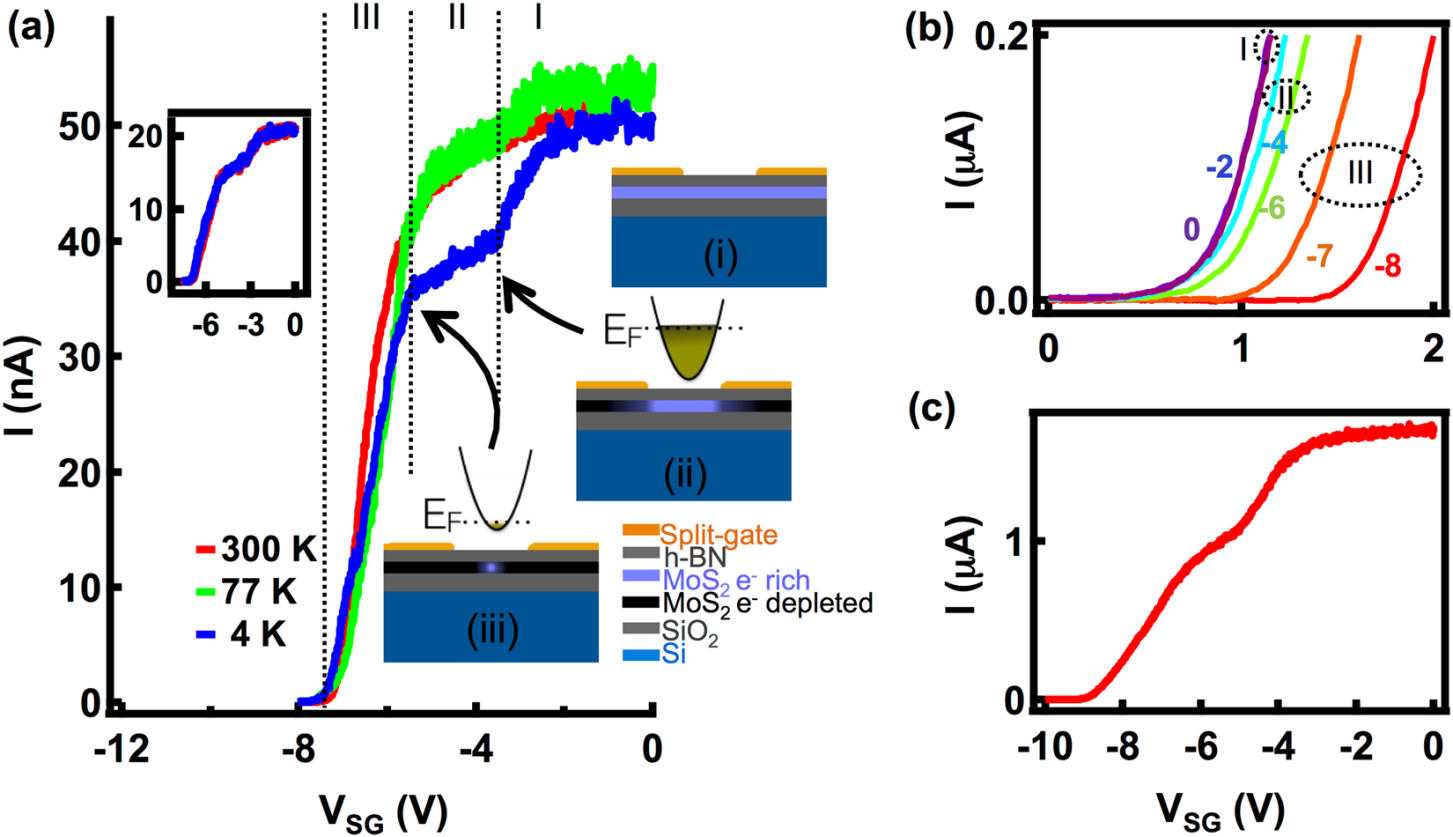
(**a**) Pinch-off characteristics at 4 K (blue), 77 K (green) and 300 K (red) where the on-state current is kept as 50 nA at V_BG_ = 10 V with illustration (i), (ii) and (iii) explaining the different regimes in the transport. The parabolas represent the electrostatic confinement of charge carriers in the point-contact. The inset shows pinch-off curves taken in the forward and the reverse direction showing no hysteresis (**b**) I-V curves as a function of V_SG_ at V_BG_ = 10 V corresponding to different regimes shown in (**a**). V_SG_ is varied from −8 V (red) to 0 V (violet), the applied V_SG_ values and the corresponding transport regimes are labelled. (**c**) Pinch-off characteristics with an on-state current of 1.7 μA showing a high on-off ratio >10^6^.

Point-contacts in our devices are realized by electrostatically modulating the conduction band in the material under the split-gate by the application of voltages, akin to the formation of those in bulk semiconductor heterostructures^28, 29^. The back-gate is used to tune the global carrier concentration in the MoS_2_ layer. For all the measurements discussed in this manuscript we maintain both the electrodes defining the split-gate at the same potential. Figure 2(a) shows the pinch-off characteristics: the current through the device as a function of the voltage on the split-gate (V_SG_), at 4 K (blue), 77 K (green) and 300 K (red). We keep an on-state current (the current through the device while the voltage on the split-gate, V_SG_ = 0 V) of 50 nA through the device and a V_BG_ of 10 V.

In contrast to the transconductance of top-gated MoS_2_ FET devices^21, 22^ the pinch-off characteristics of our split-gated devices show different regimes of transport, as shown in Fig. 2(a). In regime-I (0 V > V_SG_ > −3.6 V) the entire sample takes part in the transport (illustration-i). As V_SG_ is decreased electrons under the gate-electrodes get depleted resulting in a reduction in the current and the formation of a narrow constriction, the point contact. In this regime the transport is similar to that of a top-gated FET^21, 22^ except for the formation of the constriction. The resulting electrostatic confinement of electrons in the point contact is parabolic in nature as shown in Fig. 2(a) illustration-ii^28, 30^. In regime-II (−3.6 V > V_SG_ > −5.5 V), represented by a shoulder like feature in the pinch-off characteristics, the transport is confined through the point contact. In this regime a reduction in the V_SG_ lifts the available states in the constriction through the Fermi level causing a gradual reduction in the current and an eventual closing of the constriction. The lateral depletion of electrons in the constriction is slower compared to the depletion of electrons directly under the gate making regime-II exhibit a lower slope compared to regime-I. Once the constriction is closed the transport enters into the tunneling regime, the regime-III (illustration-iii), marked by a sharp change in the slope. A further reduction in the V_SG_ increases the barrier height and width resulting in a sharp decrease in the current and an eventual shutdown of the transport. The V_SG_ value beyond which the current measurement is limited by the noise level of the current preamplifier (~300 fA) is defined as the pinch-off voltage. The pinch-off characteristics taken at 77 K and 300 K also show all these features. The inset to Fig. 2(a) shows the pinch-off characteristics taken by sweeping V_SG_ in the forward (blue) and reverse (red) directions with an on-state current of 20 nA for V_BG_ = 10 V at 4 K. We do not observe any hysteresis effects in our device suggesting that the charge traps do not play much role in the transport behavior.

Figure 2(b) shows the two-probe I-V characteristics of the point contact device, corresponding to different transport regimes shown in Fig. 2(a), taken at 4 K, with V_BG_ = 10 V. The span of the flatter off-state region in V_DS_ in the I-V characteristics is a measure of the potential barriers involved in the transport. Here we note that the drain and source contacts turn Schottky as we lower the temperature down to 4 K, which is evident from the non-linear I-V characteristics at V_SG_ = 0 V. As V_SG_ is reduced the transport behavior smoothly changes from that of a conventional FET (regime-I) through the confined transport (regime-II) to the tunneling regime (regime-III). Both in regime-I and regime-II the only barriers involved in the transport are the drain and source contact barriers. Until the device enters the tunneling regime though the resistance of the device increases as a result of reduction in V_SG_, the turn-on voltage representing the barrier strength remains unaltered. As we move to region-III by decreasing the V_SG_ the off-state region extends further signaling the introduction of additional tunnel barrier, the point contact barrier, in the system supporting the inferences made in Fig. 2(a). Figure 2(c) shows the pinch-off characteristics of the point-contact at 4 K with an on-state current of 1.7 μA and V_BG_ = 10 V showing an on-off ratio in excess of 10^6^. The on-off ratio is estimated by taking the off-state current as the noise level of the current preamplifier. We note that by optimizing the V_BG_ and the drain-source voltage V_DS_ (not explored in this work) one can achieve much higher on-off ratios.

**Figure 3:**
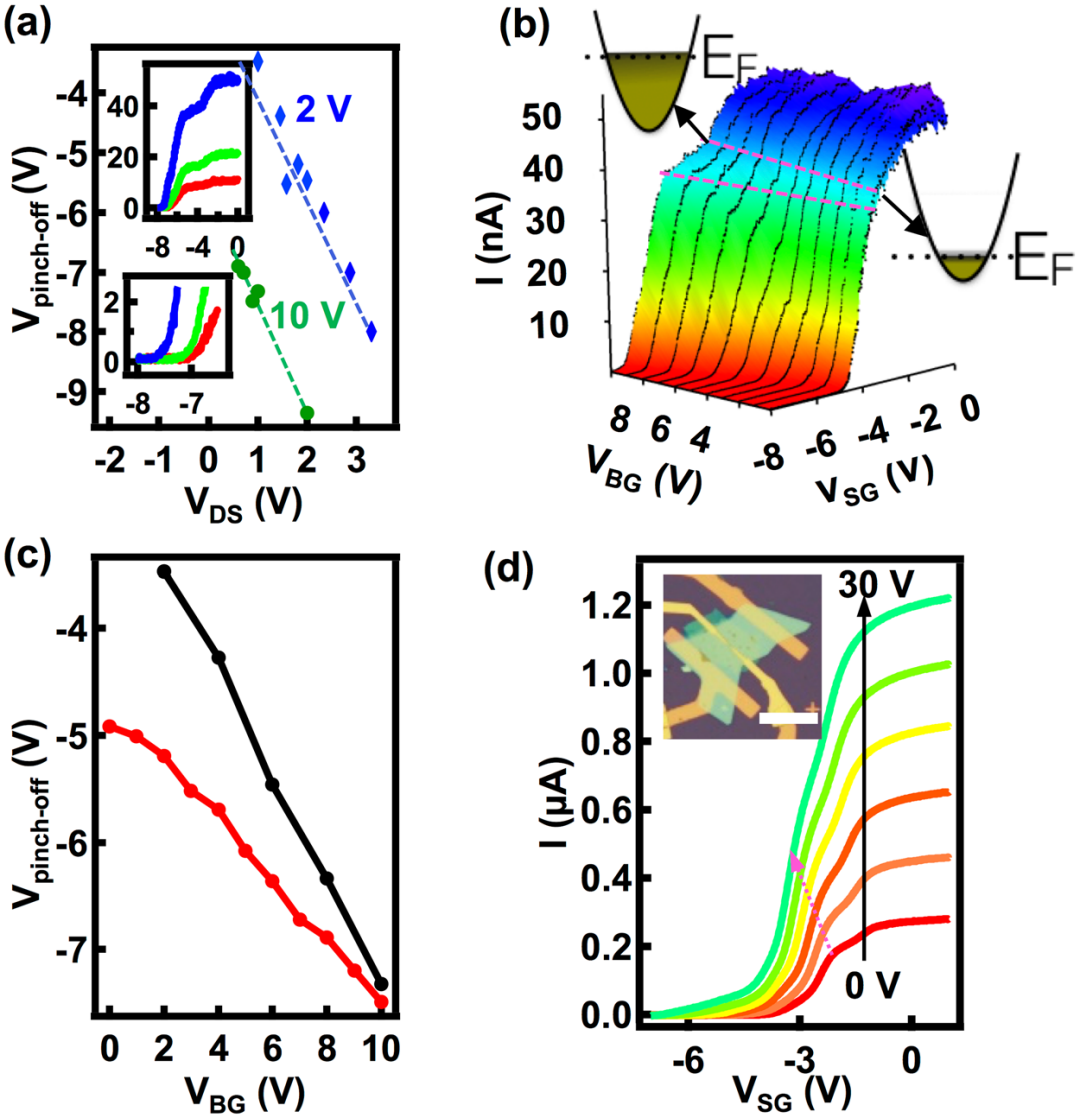
(**a**) Dependence of pinch-off voltage on the drain-source voltage for V_BG_ = 2 V (blue) and 10 V (green) at 4 K. Upper inset: pinch-off curves at three different V_DS_, 0.61 V (red), 0.72 V (green) and 0.89 V (blue) with V_BG_ = 10 V. x-axis is V_SG_ in Volts and y-axis is the current through the device in nA. Lower inset: an enlarged view of the pinch-off region. (**b**) A surface plot of pinch-off characteristics at 4 K for a series of V_BG_ with the on-state current kept as 50 nA for each trace. The regime-III, the shoulder, enclosed by the pink dashed lines show a shift in the position and a reduction in the span in V_SG_ as V_BG_ is reduced. The illustrations marked by the arrows show the available states for transport at the onset of point-contact formation for higher (V_BG_ = 10 V) and lower (V_BG_ = 0 V) carrier concentrations. (**c**) Variation of the pinch-off voltage with V_BG_. The red trace represents the pinch-off voltage extracted from (**b**), and the black trace from pinch-off characteristics taken with a V_DS_ of 1 V. (**d**) Pinch-off characteristics for another point-contact device at 300 K for various V_BG_ values. The arrow indicates the linear shift in the position of the shoulder like structure with the V_BG_. Inset: Optical image of the device, scale bar is 20 μm.

To get further insights into the pinch-off characteristics we extract the dependence of the pinch-off voltage on V_DS_ and V_BG_. Figure 3(a) shows a plot of the pinch-off voltage as a function of V_DS_ at 4 K for V_BG_ of 10 V (green) and 2 V (blue). We find that the pinch-off voltage depends linearly on V_DS_ which is a characteristic behavior of the transport across a barrier; the current through the barrier is proportional to the voltage across the barrier and the transmission coefficient of the barrier^30^. As V_DS_ is increased one need to decrease V_SG_ to shutdown the transmission by escalating the barrier. The pinch-off characteristics and an enlarged view of the pinch-off region for V_DS_ = 0.61 V (red), 0.72 V (green) and 0.89 V (blue) with V_BG_ = 10 V is shown in the top and the bottom insets respectively. The pinch-off voltage reduces as the V_DS_ is increased while the formation and closing of the point contact, represented by the shoulder region, do not strongly depend on the V_DS_ and remains unaffected.

Figure 3(b) shows a surface-plot of the pinch-off characteristics as V_BG_ is varied from 0 V through 10 V in steps of 1 V at 4 K. For all the traces, we have kept an on-state current of 50 nA by varying V_DS._ The V_DS_ values applied to maintain an on-state current of 50 nA for different back gate voltages is shown in Supplementary Information S3. All the traces exhibit structures representing the formation of the point contact and we are able to pinch-off the constriction and shutdown the transport for all V_BG_ values. The span of the shoulder region (regime-II) in V_SG_, enclosed between the two dashed lines in Fig. 3(b), decreases as V_BG_ is reduced. For a field effect device, the carrier concentration is proportional to the back-gate voltage. At lower V_BG_ the constriction has less number of states available for transport compared to higher V_BG_, as shown in the associated illustrations in Fig. 3(b). Consequently the span of V_SG_ (region-II) required to empty the states will be smaller and the depletion of electrons in the constriction and the onset of the tunneling regime will happen at a higher V_SG_ values. The complete depletion of electrons directly under the gates and the onset of formation of the point contacts also happen at higher V_SG_ values as V_BG_ is lowered. The later effect is less pronounced compared to the lateral depletion of the constriction and, as a result the span of the shoulder region in V_SG_ decreases with V_BG_. For point-contacts, the pinch-off voltage varies linearly with the carrier concentration and consequently with the back-gate voltage. An increase in the V_BG_ results in a reduction in the pinch-off voltage as observed in Fig. 3(b). An increase in the pinch-off voltage and a shrinkage of the region-II makes the pinch-off characteristics steeper at lower V_BG_ values. Figure 3(c) shows the dependence of pinch-off voltage on V_BG_. The pinch-off voltage vs V_BG_ for a constant V_DS_ of 1 V is shown in the black trace. The red trace represents the pinch-off voltages extracted from the traces in Fig. 3(b). In this case, we have increased V_DS_ as V_BG_ is reduced to keep the on-state current 50 nA for all the pinch-off curves as shown in the Supplementary Information S3. We observe that, for the same range of V_BG_ the black trace is steeper than the red trace and the red trace flattens at lower V_BG_ values. Though the reduction in the V_BG_ results in an increase in the pinch-off voltage, the corresponding increment in V_DS_ opposes it, resulting in a reduced slope compared to the black trace and an eventual flattening of the trace.

Figure 3(d) shows the room temperature pinch-off curves as a function of V_BG_ for another point-contact device made on a 20 nm thick MoS_2_ flake. An optical image of the device is shown in the inset. All pinch-off curves exhibit the shoulder-like structure and other features implying formation of point contact similar to the previous device.

## Conclusions

In this report we have demonstrated electrostatic confinement and the formation of split-gated point-contacts in a MoS_2_/h-BN heterostructures, the first step towards the realization of substrate independent quantum circuits to drive the future technology. By controlling the voltage on the split-gate we are able to control and confine the electron flow in the device leading to the formation of the point contact. The pinch-off behavior of our point contacts are similar to those realized on bulk semiconductor heterostructures. The transport in our device is differentiated from that of a through top-gated FET by the observation of different regimes in the pinch-off chaacteristics. We are able to seamlessly tune the transport across these regimes by tuning the split-gate voltage. These features are present at all temperatures ranging from 4 K to 300 K, making them potential candidates for the implementation of quantum electrical metrology and other charge detection applications at higher temperatures. We have studied the effect of carrier concentration and drain-source voltage on the pinch-off characteristics. The pinch-off voltage in our devices can be continuously tuned by varying the back-gate voltage. The devices exhibit transistor action with an on-off ratio in excess of 10^6^. The devices show nearly Ohmic behavior at room temperature, the drain and the source contacts turned non-linear at lower temperature ranges. Engineering the contacts and the dielectric interfaces would result in better devices exhibiting quantized transport.

## Electronic supplementary material

Split-gated point-contact for electrostatic confinement of transport in MoS2/h-BN hybrid structures
