# Supplementary material for: Split-gated point-contact for electrostatic confinement of transport in MoS2/h-BN hybrid structures
Source: Sci Rep. 2017 Apr 7;7:735. doi: 10.1038/s41598-017-00857-7 (PMC5429712; doi:10.1038/s41598-017-00857-7)

Supplimentary Information

Split-gated point-contact for electrostatic confinement of transport in MoS_2/h-BN hybrid structures

Chithra H. Sharma and Madhu Thalakulam*

School of Physics, Indian Institute of Science Education and Research Thiruvananthapuram

S1: Optical Image of the device

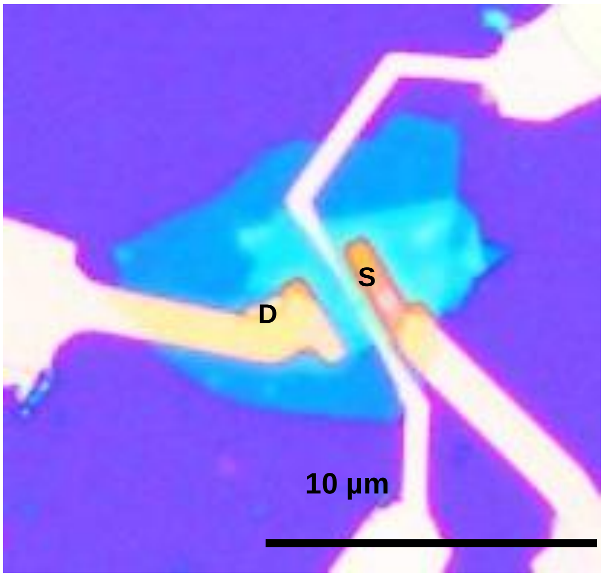

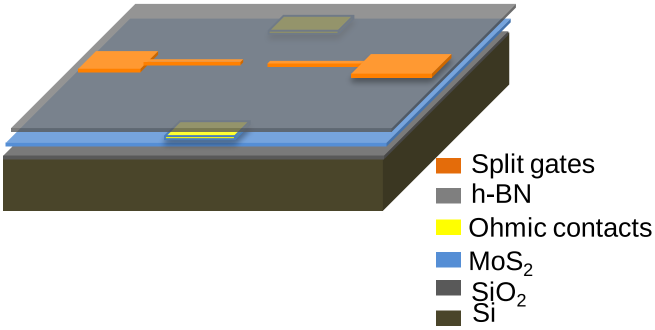

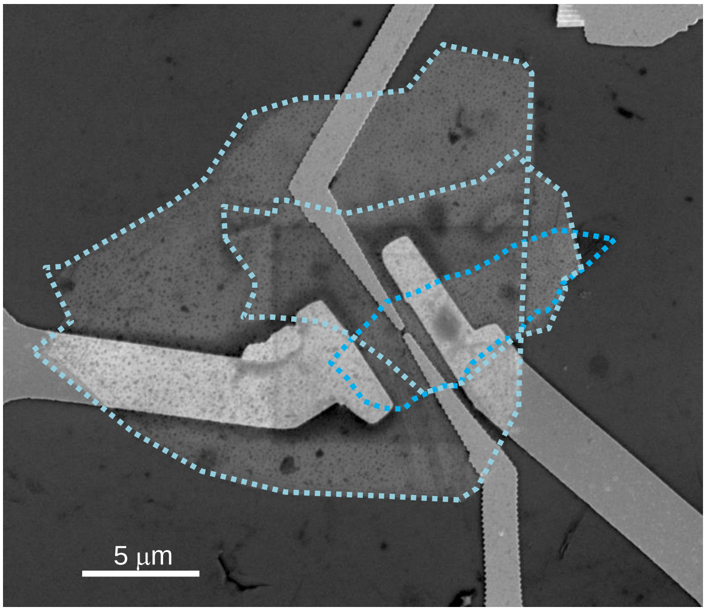

S2: AFM from which thickness of MoS_2 and h-BN flakes were obtained

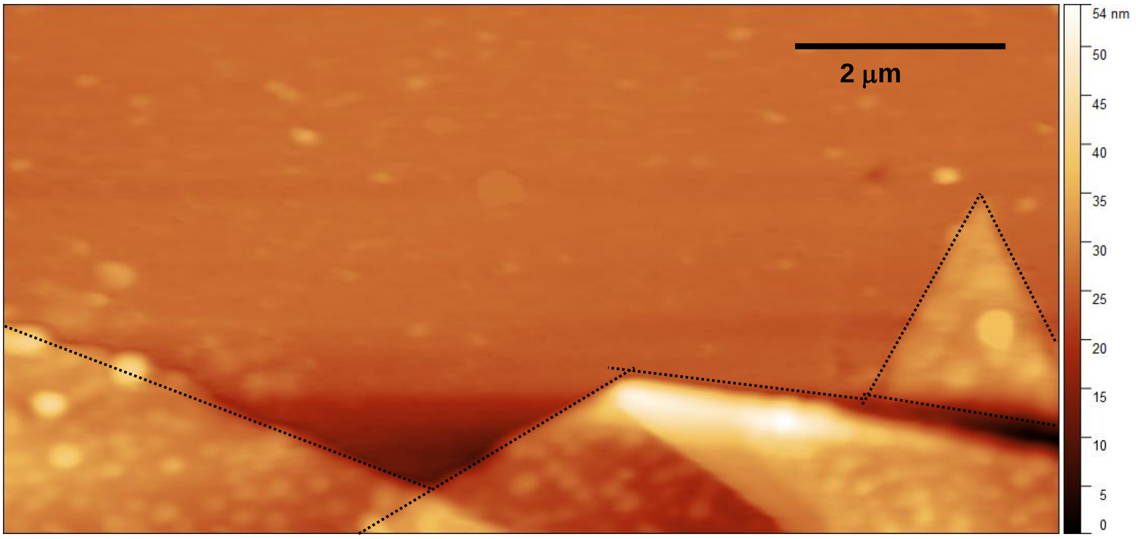

S3: Dependence of bias on back-gate voltage

The V_{DS} values used for pinch-off curves to keep the on-state current as 50 nA as the V_{BG} is varied at 4K.

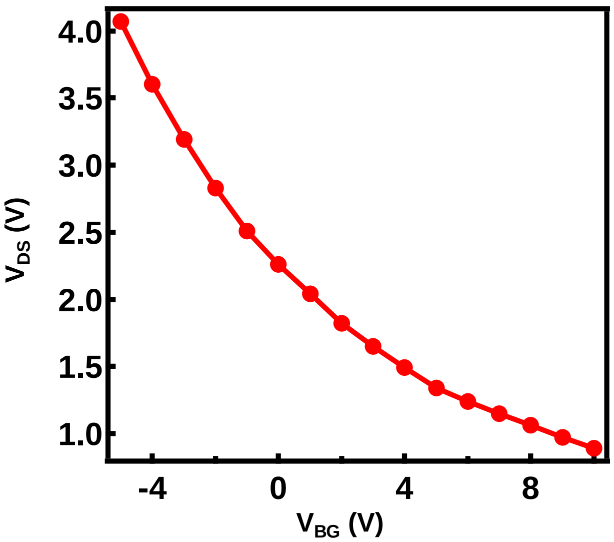

Supplement: Supplementary file 1 — Split-gated point-contact for electrostatic confinement of transport in MoS2/h-BN hybrid structures [file 41598_2017_857_MOESM1_ESM.pdf]
